# Susceptibility of ferrets, cats, dogs, and other domesticated animals to SARS–coronavirus 2

**DOI:** 10.1126/science.abb7015

**Published:** 2020-04-08

**Authors:** Jianzhong Shi, Zhiyuan Wen, Gongxun Zhong, Huanliang Yang, Chong Wang, Baoying Huang, Renqiang Liu, Xijun He, Lei Shuai, Ziruo Sun, Yubo Zhao, Peipei Liu, Libin Liang, Pengfei Cui, Jinliang Wang, Xianfeng Zhang, Yuntao Guan, Wenjie Tan, Guizhen Wu, Hualan Chen, Zhigao Bu

**Affiliations:** 1 State Key Laboratory of Veterinary Biotechnology, Harbin Veterinary Research Institute, Chinese Academy of Agricultural Sciences, Harbin 150069, People’s Republic of China.; 2 National Institute for Viral Disease Control and Prevention, China CDC, Beijing 102206, People’s Republic of China.; 3 National High Containment Laboratory for Animal Diseases Control and Prevention, Harbin 150069, People’s Republic of China.

## Abstract

Severe acute respiratory syndrome–coronavirus 2 (SARS-CoV-2) causes the infectious disease COVID-19 (coronavirus disease 2019), which was first reported in Wuhan, China, in December 2019. Despite extensive efforts to control the disease, COVID-19 has now spread to more than 100 countries and caused a global pandemic. SARS-CoV-2 is thought to have originated in bats; however, the intermediate animal sources of the virus are unknown. In this study, we investigated the susceptibility of ferrets and animals in close contact with humans to SARS-CoV-2. We found that SARS-CoV-2 replicates poorly in dogs, pigs, chickens, and ducks, but ferrets and cats are permissive to infection. Additionally, cats are susceptible to airborne transmission. Our study provides insights into the animal models for SARS-CoV-2 and animal management for COVID-19 control.

## Alternative hosts and model animals

The severe acute respiratory syndrome–coronavirus 2 (SARS-CoV-2) pandemic may have originated in bats, but how it made its way into humans is unknown. Because of its zoonotic origins, SARS-CoV-2 is unlikely to exclusively infect humans, so it would be valuable to have an animal model for drug and vaccine development. Shi *et al.* tested ferrets, as well as livestock and companion animals of humans, for their susceptibility to SARS-CoV-2 (see the Perspective by Lakdawala and Menachery). The authors found that SARS-CoV-2 infects the upper respiratory tracts of ferrets but is poorly transmissible between individuals. In cats, the virus replicated in the nose and throat and caused inflammatory pathology deeper in the respiratory tract, and airborne transmission did occur between pairs of cats. Dogs appeared not to support viral replication well and had low susceptibility to the virus, and pigs, chickens, and ducks were not susceptible to SARS-CoV-2.

*Science*, this issue p. 1016; see also p. 942

## Teaser

Ferrets and cats are permissive to SARS-CoV-2 replication and show respiratory tract pathology.

In late December 2019, an unusual pneumonia emerged in humans in Wuhan, China, and rapidly spread internationally, raising global public health concerns. The causative pathogen was identified as a novel coronavirus (*1*–*16*) and named severe acute respiratory syndrome–coronavirus 2 (SARS-CoV-2) on the basis of a phylogenetic analysis of related coronaviruses by the Coronaviridae Study Group of the International Committee on Taxonomy of Viruses (*17*). Subsequently, the disease caused by this virus was designated coronavirus disease 2019 (COVID-19) by the World Health Organization (WHO). Despite major efforts to control the COVID-19 outbreak, the disease is still spreading. As of 11 March 2020, SARS-CoV-2 infections have been reported in more than 100 countries, and 118,326 human cases have been confirmed, with 4292 fatalities (*18*). WHO has now officially declared COVID-19 a pandemic.

Although SARS-CoV-2 shares 96.2% of its identity at the nucleotide level with the coronavirus RaTG13—which was detected in horseshoe bats (*Rhinolophus* spp.) in Yunnan province, China, in 2013 (*3*)—it has not previously been detected in humans or other animals. The emerging public health crisis raises many urgent questions. Could the widely disseminated SARS-CoV-2 be transmitted to other animal species, which then become reservoirs of infection? The SARS-CoV-2 infection has a wide clinical spectrum in humans, ranging from mild infection to death, but how does the virus behave in other animals? As efforts progress toward vaccine and antiviral drug development, which animal(s) can be used to most accurately model the efficacy of such control measures in humans? To address these questions, we evaluated the susceptibility of different model laboratory animals, as well as companion and domestic animals, to SARS-CoV-2.

All experiments with infectious SARS-CoV-2 were performed in the biosafety level 4 and animal biosafety level 4 facilities in the Harbin Veterinary Research Institute (HVRI) of the Chinese Academy of Agricultural Sciences (CAAS), which was approved for such use by the Ministry of Agriculture and Rural Affairs of China. Details of the biosafety and biosecurity measures are provided in the supplementary materials (*19*). The protocols for animal study and animal welfare were reviewed and approved by the Committee on the Ethics of Animal Experiments of the HVRI of CAAS (approval number 2020-01-01JiPi).

**Fig. 1:**
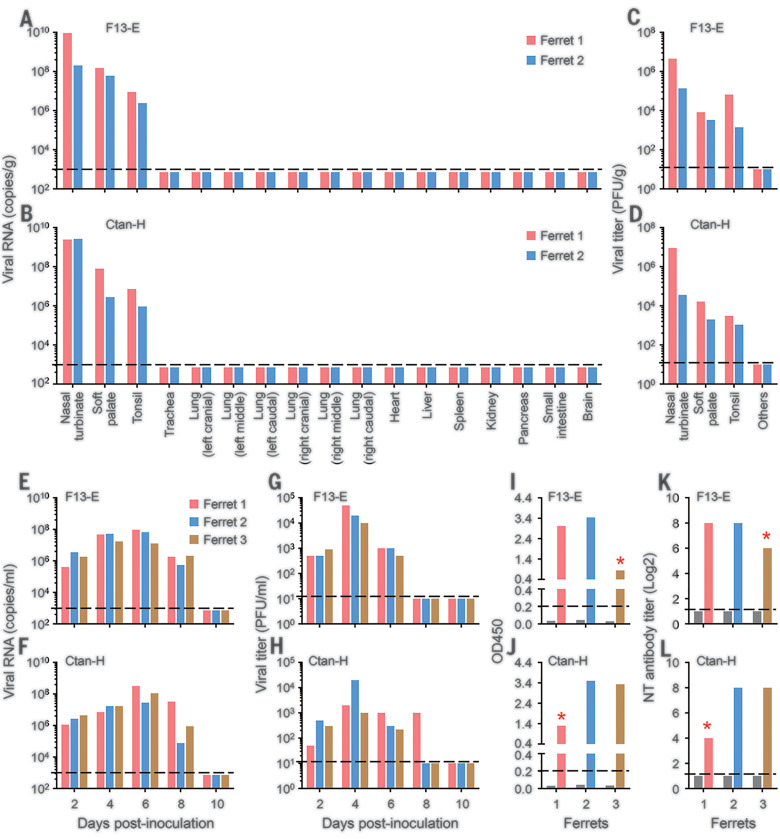
Replication of SARS-CoV-2 in ferrets. Viral RNA in organs or tissues of ferrets inoculated with (**A**) F13-E virus strain or (**B**) CTan-H strain. Viral titers in organs or tissues of ferrets inoculated with F13-E (**C**) and CTan-H (**D**). The viral RNA–negative organs indicted in (A) and (B) were also negative for virus titration [indicated as “Others” in (C) and (D)]. Viral RNA (**E** and **F**) and viral titer (**G** and **H**) in nasal washes of ferrets inoculated with F13-E [(E) and (G)] and CTan-H [(F) and (H)]. Antibodies against SARS-CoV-2 tested by ELISA (**I** and **J**) and neutralization assay (**K** and **L**) with sera derived from ferrets inoculated with F13-E [(I) and (K)] and CTan-H [(J) and (L)]. Each color bar represents the value from an individual animal. The gray bars in (I) to (L) indicate the antibody values of sera collected from each animal before inoculation. Asterisks denote animals that were euthanized on day 13 p.i.; the other four animals were euthanized on day 20 p.i. The dashed lines in (I) and (L) show the cutoff value for seroconversion, and the dashed lines in the other panels indicate the lower limit of detection. OD450, optical density measured at 450 nm; NT antibody, neutralizing antibody.

Ferrets are commonly used as an animal model for viral respiratory infections in humans (*20*–*26*). We therefore tested the susceptibility of ferrets to SARS-CoV-2. Two virus strains were used in this study: (i) SARS-CoV-2/F13/environment/2020/Wuhan (F13-E), isolated from an environmental sample collected in the Huanan Seafood Market in Wuhan, and (ii) SARS-CoV-2/CTan/human/2020/Wuhan (CTan-H), isolated from a human patient. Pairs of ferrets were inoculated intranasally with 10^5^ plaque-forming units (PFU) of F13-E or CTan-H and euthanized on day 4 postinoculation (p.i.). The nasal turbinate, soft palate, tonsils, trachea, lung, heart, liver, spleen, kidneys, pancreas, small intestine, and brain from each ferret were collected for viral RNA quantification by quantitative polymerase chain reaction and virus titration in Vero E6 cells. Viral RNA (Fig. 1, A and B) and infectious virus (Fig. 1, C and D) were detected in the nasal turbinate, soft palate, and tonsils of all four ferrets inoculated with these two viruses but were not detected in any other organs tested. These results indicate that SARS-CoV-2 can replicate in the upper respiratory tract of ferrets, but its replication in other organs is undetectable.

To investigate the replication dynamics of these virus strains in ferrets, groups of three animals were inoculated intranasally with 10^5^ PFU of F13-E or CTan-H, and each ferret was then placed in a separate cage within an isolator. Nasal washes and rectal swabs were collected on days 2, 4, 6, 8, and 10 p.i. from the ferrets for viral RNA detection and virus titration. Body temperatures and signs of disease were monitored for 2 weeks. Viral RNA was detected in the nasal washes on days 2, 4, 6, and 8 p.i. in all six ferrets inoculated with the two strains (Fig. 1, E and F). Viral RNA was also detected in some of the rectal swabs of the virus-inoculated ferrets, although the copy numbers were notably lower than those in the nasal washes of these ferrets (fig. S1, A and C). Infectious virus was detected from the nasal washes of all ferrets (Fig. 1, G and H) but not from the rectal swabs of any ferrets (fig. S1, B and D).

One ferret from each virus-inoculated group developed fever and loss of appetite on days 10 (CTan-H–inoculated) and 12 p.i. (F13-E–inoculated), respectively. To investigate whether these symptoms were caused by virus replication in the lower respiratory tract, we euthanized the two ferrets on day 13 p.i. and collected their organs for viral RNA detection. However, viral RNA was not detected in any other tissues or organs of either ferret, except for a low copy number (10^5.4^ copies/g) in the turbinate of the ferret inoculated with CTan-H (fig. S2). Pathological studies revealed severe lymphoplasmacytic perivasculitis and vasculitis; increased numbers of type II pneumocytes, macrophages, and neutrophils in the alveolar septa and alveolar lumen; and mild peribronchitis in the lungs of the two ferrets euthanized on day 13 p.i. (fig. S3). Antibodies against SARS-CoV-2 were detected in all ferrets by an enzyme-linked immunosorbent assay (ELISA) and a neutralization assay, although the antibody titers of the two ferrets that were euthanized on day 13 p.i. were notably lower than those of the ferrets euthanized on day 20 p.i. (Fig. 1, I to L).

A virus attachment assay indicated that SARS-CoV-2 could attach to bronchiolar epithelial cells (fig. S4A) and some type II pneumocytes (fig. S4B) in ferret lungs. To further investigate whether SARS-CoV-2 replicates in the lungs of ferrets, we intratracheally inoculated eight ferrets with 10^5^ PFU of CTan-H and euthanized two animals each on days 2, 4, 8, and 14 p.i. to look for viral RNA in the tissues and organs. Viral RNA was detected only in the nasal turbinate and soft palate of one ferret in each pair euthanized on days 2 and 4 p.i.; was detected in the soft palate of one ferret and in the nasal turbinate, soft palate, tonsils, and trachea of the other ferret euthanized on day 8 p.i.; and was not detected in either of the two ferrets euthanized on day 14 p.i. (fig. S5). These results indicate that SARS-CoV-2 can replicate in the upper respiratory tract of ferrets for up to 8 days without causing severe disease or death.

Cats and dogs are in close contact with humans; therefore, it is important to understand their susceptibility to SARS-CoV-2. We first investigated the replication of SARS-CoV-2 in cats. Seven subadult cats (aged 6 to 9 months, outbred domestic cats) were intranasally inoculated with 10^5^ PFU of CTan-H. Two animals were scheduled to be euthanized on days 3 and 6 p.i., respectively, to evaluate viral replication in their organs. Three subadult cats were placed in separate cages within an isolator. To monitor respiratory droplet transmission, an uninfected cat was placed in a cage adjacent to each of the infected cats. The aggressive behavior of the subadult cats made it difficult to perform regular nasal wash collection. To avoid possible injury, we only collected feces from these cats and checked for viral RNA in their organs after euthanasia.

**Fig. 2:**
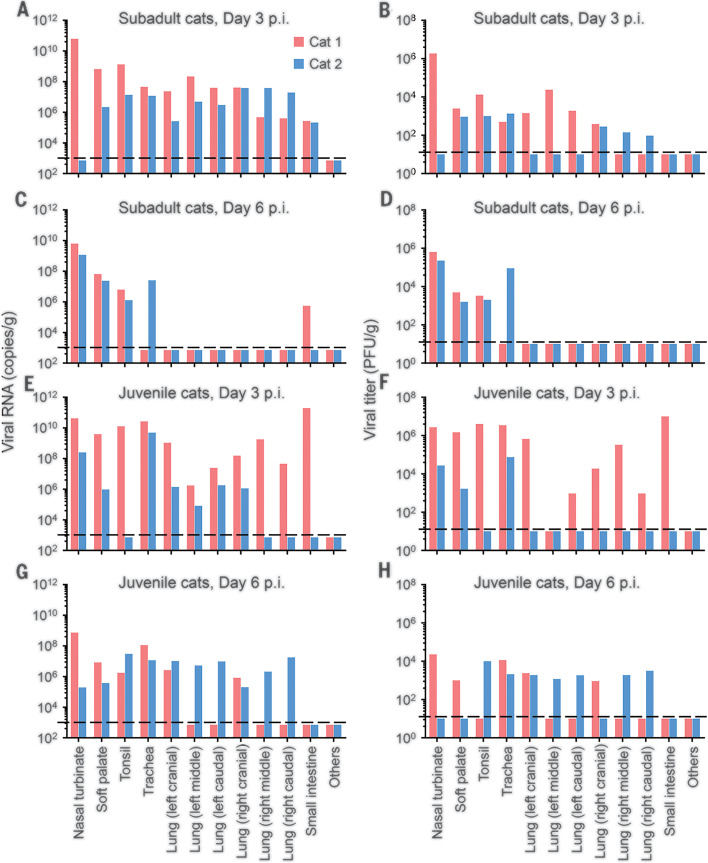
Replication of SARS-CoV-2 in cats. Subadult cats and juvenile cats inoculated with CTan-H virus were euthanized on days 3 and 6 p.i., and their organs were collected for viral RNA detection and virus titration. (**A**) Viral RNA and (**B**) viral titers of subadult cats on day 3 p.i. (**C**) Viral RNA and (**D**) viral titers of subadult cats on day 6 p.i. (**E**) Viral RNA and (**F**) viral titers of juvenile cats on day 3 p.i. The values of red bars in (E) and (F) are from the cat that died on this day. (**G**) Viral RNA and (**H**) viral titers of juvenile cats on day 6 p.i. “Others” represents viral-negative organs, including the brain, heart, submaxillary lymph nodes, kidneys, spleen, liver, and pancreas. Each color bar represents the value from an individual animal. The dashed lines indicate the lower limit of detection.

Viral RNA was detected in the nasal turbinate of one animal, as well as in the soft palates, tonsils, tracheas, lungs, and small intestines of both animals euthanized on day 3 p.i. (Fig. 2A). In the animals euthanized on day 6 p.i., viral RNA was detected in the nasal turbinates, soft palates, and tonsils of both animals; in the trachea of one animal; and in the small intestine of the other. However, viral RNA was not detected in any lung samples from either of these animals (Fig. 2C). Infectious virus was detected in the viral RNA–positive nasal turbinates, soft palates, tonsils, tracheas, and lungs of these cats but was not recovered from the viral RNA–positive small intestines (Fig. 2, B and D)

**Fig. 3:**
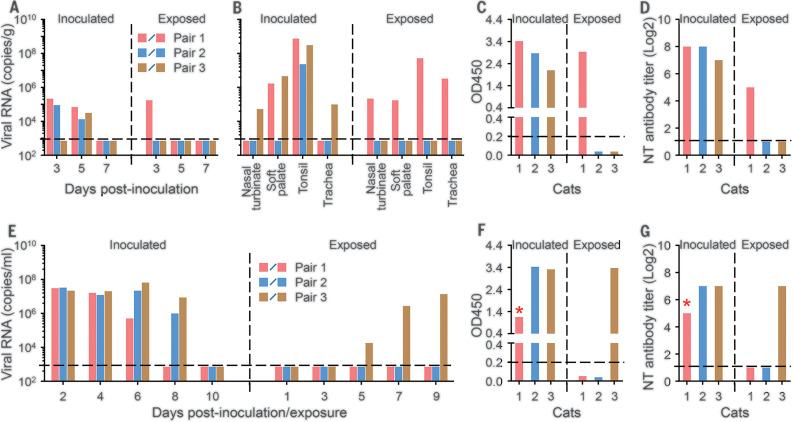
Transmission of SARS-CoV-2 in cats. Transmission of the CTan-H virus strain was evaluated in subadult cats (**A** to **D**) and juvenile cats (**E** to **G**). (A) Viral RNA in the feces of virus-inoculated or virus-exposed subadult cats. (B) Viral RNA in tissues or organs of inoculated or exposed subadult cats euthanized on day 11 p.i. (pair 1, red bars) or day 12 p.i. (pairs 2 and 3). Antibodies against SARS-CoV-2 in these euthanized subadult cats were detected by ELISA (C) and neutralization assay (D). (E) Viral RNA in nasal washes of juvenile cats. Sera from juvenile cats were collected on day 20 p.i., except for sera from one virus-inoculated animal that died on day 13 p.i. Antibody values for this cat (indicated by asterisks) were detected from sera collected on day 10 p.i.; antibodies against SARS-CoV-2 were detected by ELISA (F) and neutralization assay (G). Each color bar represents the value from an individual animal. The horizontal dashed lines in (C) and (F) show the cutoff value for seroconversion, and the horizontal dashed lines in the other panels indicate the lower limit of detection.

In the transmission study, viral RNA was detected in the feces of two virus-inoculated subadult cats on day 3 p.i. and in all three virus-inoculated subadult cats on day 5 p.i. (Fig. 3A). Viral RNA was detected in the feces of one exposed cat on day 3 p.i. (Fig. 3A). The pair of subadult cats with viral RNA–positive feces was euthanized on day 11 p.i. Viral RNA was detected in the soft palate and tonsils of the virus-inoculated animal and in the nasal turbinate, soft palate, tonsils, and trachea of the exposed animal (Fig. 3B), indicating that respiratory droplet transmission had occurred. We euthanized the other pairs on day 12 p.i. Viral RNA was detected in the tonsils of one virus-inoculated subadult cat and in the nasal turbinate, soft palate, tonsils, and trachea of the other virus-inoculated subadult cat but was not detected in any organs or tissues of the two exposed subadult cats (Fig. 3B). Antibodies against SARS-CoV-2 were detected in all three virus-inoculated subadult cats and one exposed cat via an ELISA and neutralization assay (Fig. 3, C and D).

We repeated the replication and transmission studies in juvenile cats (aged 70 to 100 days) (Figs. 2, E to H, and 3, E to G, and fig. S6). Histopathologic studies performed on samples from the virus-inoculated juvenile cats that died or were euthanized on day 3 p.i. revealed massive lesions in the nasal and tracheal mucosa epitheliums and lungs (fig. S7). These results indicate that SARS-CoV-2 can replicate efficiently in cats and that younger cats are more vulnerable than older ones. Notably, our findings also reveal that the virus is transmissible between cats via the airborne route.

We next investigated the replication and transmission of SARS-CoV-2 in dogs. Five 3-month-old beagles were intranasally inoculated with 10^5^ PFU of CTan-H and housed with two uninoculated beagles in a room. Oropharyngeal and rectal swabs from each beagle were collected on days 2, 4, 6, 8, 10, 12, and 14 p.i. for viral RNA detection and virus titration in Vero E6 cells. Viral RNA was detected in the rectal swabs of two virus-inoculated dogs on day 2 p.i. and in the rectal swab of one such dog on day 6 p.i. (Table 1). One dog that was positive for viral RNA by rectal swab on day 2 p.i. was euthanized on day 4 p.i., but viral RNA was not detected in any organs or tissues collected from this animal (fig. S8). Additionally, infectious virus was not detected in any swabs collected from any of these dogs. Sera were collected from all dogs on day 14 p.i. for antibody detection by an ELISA. Two virus-inoculated dogs seroconverted; the other two virus-inoculated dogs and the two contact-exposed dogs were all seronegative for SARS-CoV-2 (Table 1 and fig. S9). These results indicate that dogs have low susceptibility to SARS-CoV-2.

**Table 1:** Susceptibility of dogs, pigs, chickens, and ducks to SARS-CoV-2. Animals were intranasally inoculated with 10^5^ PFU (dogs and pigs) or 10^4.5^ PFU (chickens and ducks) of the CTan-H virus strain. Two (dogs) or three (pigs, chickens, and ducks) uninfected animals were housed in the same room with their infected counterparts to monitor transmission of the virus. Oropharyngeal and rectal swabs from all animals were collected on the indicated days postinoculation (p.i.) for viral RNA detection. The “Other time points” category includes days 8, 10, 12, and 14 p.i.

**Animal**	**Treatment**	**Viral RNA detection in animals inoculated with SARS-CoV-2 isolate CTan-H;** **positive cases/total (copies, log_10_)**								**Seroconversion;** **positive** **cases/total†**
		**Oropharyngeal swab**				**Rectal swab**				
		**Day 2 p.i.**	**Day 4 p.i.**	**Day 6 p.i.**	**Other time** **points**	**Day 2 p.i.**	**Day 4 p.i.**	**Day 6 p.i.**	**Other time** **points**	
Dog*	Inoculated	0/5	0/5	0/4	0/4	2/5 (6.5, 5.4)	0/5	1/4 (4.2)	0/4	2/4
	Contact	0/2	0/2	0/2	0/2	0/2	0/2	0/2	0/2	0/2
Pig	Inoculated	0/5	0/5	0/5	0/5	0/5	0/5	0/5	0/5	0/5
	Contact	0/3	0/3	0/3	0/3	0/3	0/3	0/3	0/3	0/3
Chicken	Inoculated	0/5	0/5	0/5	0/5	0/5	0/5	0/5	0/5	0/5
	Contact	0/3	0/3	0/3	0/3	0/3	0/3	0/3	0/3	0/3
Duck	Inoculated	0/5	0/5	0/5	0/5	0/5	0/5	0/5	0/5	0/5
	Contact	0/3	0/3	0/3	0/3	0/3	0/3	0/3	0/3	0/3

*One virus-inoculated beagle was euthanized on day 4 p.i., but viral RNA was not detected in any of its collected organs, which included lung, trachea, nasal turbinate, soft palate, brain, heart, tonsils, kidneys, spleen, liver, pancreas, and small intestine (fig. S6).

†Sera were collected from all animals on day 14 p.i., and antibodies against SARS-CoV-2 were detected by using a double-antigen sandwich ELISA kit (ProtTech, Luoyang, China).

We also investigated the susceptibility of pigs, chickens, and ducks to SARS-CoV-2 by using the same strategy as that used to assess dogs. However, viral RNA was not detected in any swabs collected from these virus-inoculated animals or from naïve contact animals (Table 1). In addition, all of these animals were seronegative for SARS-CoV-2 when tested by ELISA with sera collected on day 14 p.i. (Table 1). These results indicate that pigs, chickens, and ducks are not susceptible to SARS-CoV-2.

In summary, we found that ferrets and cats are highly susceptible to SARS-CoV-2; dogs have low susceptibility; and pigs, chickens, and ducks are not susceptible to the virus. Unlike influenza viruses and the other SARS-coronavirus known to infect humans (SARS-CoV-1), which replicate in both the upper and lower respiratory tract of ferrets (*20*, *22*–*24*, *26*, *27*), SARS-CoV-2 replicates only in the nasal turbinate, soft palate, and tonsils of ferrets. SARS-CoV-2 may also replicate in the digestive tract, as viral RNA was detected in the rectal swabs of the virus-infected ferrets, but virus was not detected in lung lobes, even after the ferrets were intratracheally inoculated with the virus. It remains unclear whether the virus causes more severe disease in male ferrets than in female ferrets, as has been observed among humans (*13*, *28*).

Several studies have reported that SARS-CoV-2 uses angiotensin-converting enzyme 2 (ACE2) as its receptor to enter cells (*3*, *29*–*31*). ACE2 is mainly expressed in type II pneumocytes and serous epithelial cells of tracheo-bronchial submucosal glands in ferrets (*25*). Ferrets and cats differ by only two amino acids in the SARS-CoV-2 spike-contacting regions of ACE2 (table S1); therefore, the underlying mechanism that prevents the replication of SARS-CoV-2 in the lower respiratory tract of ferrets remains to be investigated. The fact that SARS-CoV-2 replicates efficiently in the upper respiratory tract of ferrets makes them a candidate animal model for evaluating the efficacy of antiviral drugs or vaccines against COVID-19.

The cats we used in this study were outbred and were susceptible to SARS-CoV-2, which replicated efficiently and was transmissible to naïve cats. Cats in Wuhan have been reported to be seropositive for SARS-CoV-2 (*32*). Surveillance for SARS-CoV-2 in cats should be considered as an adjunct to elimination of COVID-19 in humans.

## Supplementary Materials

science.sciencemag.org/content/368/6494/1016/suppl/DC1 Materials and Methods Supplementary Text Figs. S1 to S10 Table S1 References and Notes (*33*–*35*) MDAR Reproducibility Checklist

## Appendix

View/request a protocol for this paper from *Bio-protocol*.
